# Fitter Fontans for future—Impact of physical exercise on cardiopulmonary function in Fontan patients

**DOI:** 10.3389/fcvm.2022.972652

**Published:** 2022-09-12

**Authors:** Annika Weigelt, Regina Fritsch, Kathrin Rottermann, Wolfgang Wällisch, Julia Moosmann, Sven Dittrich, Ariawan Purbojo, Isabelle Schöffl

**Affiliations:** ^1^Department of Pediatric Cardiology, Friedrich-Alexander-Universität Erlangen-Nürnberg, Erlangen, Germany; ^2^Department of Pediatric Heart Surgery, Friedrich-Alexander-Universität Erlangen-Nürnberg, Erlangen, Germany; ^3^School of Clinical and Applied Sciences, Leeds Beckett University, Leeds, United Kingdom

**Keywords:** *V̇O*_2peak_, peak oxygen uptake, cardiopulmonary exercise testing (CPET), congenital heart disease, sports and exercise medicine, TCPC, habitual exercise

## Abstract

**Background:**

In Fontans, exercise tolerance is poorer compared to their healthy peers. Higher $\dot{V}O_{2}peak$ represents a strong predictor for mortality and morbidity in these patients. Cardiac rehabilitation programs have been shown to improve cardiopulmonary function in Fontans. More habitual physical activity should therefore lead to a better exercise tolerance.

**Methods:**

We performed cardiopulmonary exercise testing in 24 Fontan patients who had engaged in physical activity for a minimum of 3 h per week over their lifetime. As a control we performed cardiopulmonary exercise testing in 20 Fontan patients who had undertaken no physical activity or <3 h per week in the past.

**Results:**

A total of 44 Fontan patients was included (mean age 18.1 years). The mean parameters measured at peak exercise differed significantly between the active and inactive group (peak oxygen uptake [$\dot{V}O_{2}peak$] of 34.0 vs. 25.0 ml/min/kg, peak heart rate (HR) of 169.8/min vs. 139.8/min). Even though the O_2_pulse and the EF did not differ significantly between both groups, N-Terminal-Pro-B-Type Natriuretic Peptide (NT-pro BNP) was significantly higher in the inactive group. The two groups did not differ with respect to their cardiac function determined by magnetic resonance imaging (MRI). $\dot{V}O_{2}peak$ was positively correlated with hours of sports performed by Fontans.

**Conclusions:**

$\dot{V}O_{2}peak$ and maximum HR were significantly higher in Fontans who had been physically active compared to those who had been inactive. The values reported in this study were higher than in other studies and reached normal values for $\dot{V}O_{2}peak$ for most Fontans in the physically active group. The positive correlation between $\dot{V}O_{2}peak$ and physical activity is an indicator of the importance of incorporating physical exercise programs into the treatment of Fontan patients.

## Introduction

Even though the majority of patients with Fontan circulation reach adulthood (1), morbidity and mortality remain high (2).

There is a strong negative association between $\dot{V}O_{2}peak$ (peak oxygen uptake) morbidity, and mortality (3). $\dot{V}O_{2}peak$ is generally used for characterizing exercise capacity (4). Even after adjusting for heart failure according to the classification presented by the New York Heart Association (NYHA-classification), institution, and Fontan type, the severity of Fontan associated liver disease is higher if $\dot{V}O_{2}peak\;$is reduced (5). There is a stronger correlation between $\dot{V}O_{2}peak\;$and quality of life (QoL) compared to echocardiographic parameters and QoL (6). Another strong predictor for mortality and transplantation in Fontan patients is peak heart rate (HR) during cardiopulmonary exercise testing (3). The impaired ability to reach target HR in Fontan patients is due to premature reductions in ventricular filling and stroke volume limiting further increases in HR as cardiac output plateaus (7, 8). Generalized, a greater exercise capacity characterizes good Fontan survivors (9).

On average, Fontan patients reach a $\dot{V}O_{2}peak$ of about 65% of their predicted value with a progressive decline of about 2.6% per year (8, 10). This exercise impairment may be due to central cardiovascular factors (oxygen delivery) as well as peripheral skeletal muscle factors (oxygen extraction) (8, 11). The central cardiovascular factors cannot easily be improved (8), whereas peripheral factors, especially muscle mass and fitness could represent modifiable parameters for improving $\dot{V}O_{2}peak$ in Fontan patients (8, 11). However, patients after Fontan repair are more sedentary than their healthy peers (12) and do not achieve daily levels of moderate to vigorous exercise as recommended (13). This is often a consequence of parents who are uncertain about the recommended physical activity of their children (13).

Several studies have been able to show the positive impact of physical exercise on $\dot{V}O_{2}peak$ in Fontan patients (14–16). However, exercise programs focus on a limited time period of physical intervention and thus, do not reflect the overall impact of an active lifestyle on exercise capacity in Fontan patients. Furthermore, a positive exercise capacity trajectory during childhood, defined as a rise of $\dot{V}O_{2}peak$ over time, led to better exercise capacity, hemodynamics and pulmonary function in adult Fontan patients and as a consequence, to a better adult Fontan physiology. A positive post-childhood exercise capacity trajectory did not yield the same positive results (17) which stresses the importance of early and ongoing involvement of physical exercise in Fontan patients.

So far, the influence of habitual physical exercise, as performed in extracurricular sports club activities, on exercise tolerance after Fontan procedure, has only been investigated by Kodama et al. (14). They concluded that changing exercise habits could promote exercise tolerance in Fontan patients (14). However, their observation was limited to a young cohort (12.8 ± 3.3 y) and long-term effects of habitual physical exercise have not been elucidated.

Because of these promising results, we wanted to investigate the long-term effect of physical activity and sports throughout childhood and adolescence on exercise parameters in patients after Fontan palliation.

## Materials and methods

The study was approved by the Ethics Committee of the University of Erlangen-Nuremberg, FRG (95_19B).

We evaluated diagnostic data and surgery-related characteristics (ie, number of previous surgeries and complications) of 44 patients with Fontan circulation which were extracted from the patients' hospital records and database. The participants received their palliation procedure at our university hospital, were older than 4 years of age and performed a cardiopulmonary exercise testing (CPET) within the last 3 years. All study participants as well as their legal guardians, if indicated, gave informed consent.

We used a non-standardized questionnaire to determine school or work transport habits (walking, cycling, bus), and subjective exercise tolerance. With regards to subjective exercise tolerance, the subjects were asked whether they felt as tolerant to exercise as their healthy, age-matched peers or less so. This subjective parameter was used for assessing how the subjects perceived themselves with regards to their physical fitness. As such it does not represent a standardized parameter but is meant to reflect satisfaction with one's fitness level. We did not use the NYHA scale as it is not sufficiently validated in adolescents and only assesses limitation with respect to physical exercise and does not allow for comparison with healthy controls. Additionally, the questionnaire evaluated the amount of extracurricular vigorous physical activity performed by each participant as a preschooler (3–6 years of age), during elementary school (7–10 years of age), as an adolescent (11–18 years of age) and recently performed, in hours per week. Vigorous activity was classified as requiring a significant increase of HR, e.g., running, trampolining, playing sports or participating in club sports. According to the recommendation for physical activity in adults by the WHO (18), we divided the patients into two groups: those who undertook a minimum of 3 h of vigorous physical activity at any time during their lives (between preschool and present time) and those who had never performed vigorous sports for a minimum of 3 h a week.

Height and weight were measured using a stadiometer and electronic scale (Seca 704 S, Hamburg, Germany). For children and adolescents z-scores were determined using measurements from the German Health Interview and Examination Survey for Children and Adolescents (KIGGS-survey) (19).

Medical records were reviewed for information about the underlying cardiac disease, complications, previous surgeries, and current medications. We also included data from magnetic resonance imaging (MRI) examinations if the time between MRI and cardiopulmonary exercise test was no longer than 4 years. The following associated hemodynamic abnormalities identified by MRI were listed with their severity: aortic/neoaortic regurgitation, atrioventricular valve regurgitation (AV valve regurgitation), aortic/neoaortic arch obstruction and presence of aorto-pulmonary collaterals. The severity of regurgitation was recorded as regurgitant fraction which was defined as the backflowing blood volume divided by the forward flow volume in percent [retrograde flow volume (ml)/antegrade flow volume (ml)]. Tidal volume as an index of respiratory function was recorded prior to CPET.

### Measurement of gas exchange

A small, low-dead-space respiratory valve (88 ml) with a size-matched mouthpiece and headgear was used (Metalyzer 3B, Cortex, Leipzig, Germany). Gas-exchange was measured continuously during each test using a breath-by-breath method and averaged over 15 s intervals. The highest $\dot{V}O_{2}$ recorded during a single graded exercise test can be used as an estimate of $\dot{V}O_{2}peak$ (20). $\dot{V}O_{2}peak$ should not be construed with $\dot{V}O_{2\;}\max$, which represents the maximum rate of oxygen uptake and is difficult to accomplish in practice (20). In clinical populations, differences between $\dot{V}O_{2}peak$ and mode-specific $\dot{V}O_{2\;\max}$ are likely to be small when appropriate quality control is applied (20). We therefore used the following physiological criteria for completion of a valid $\dot{V}O_{2}peak$, two of which needed to be met for validation: (1) peak HR within 5% of the age-predicted maximum, (2) respiratory exchange ratio (RER) ≥1.0, and (3) volitional fatigue (21, 22). The first ventilatory threshold (VT1) is a marker of intensity that can be observed in a person's breathing at a point where lactate begins to accumulate in the blood. VT_1_ was determined according to the modified V-slope method in combination with the ventilatory equivalent method ($\dot{V}E/VO_{2}\;=$ minute ventilation/oxygen uptake) and the end-tidal O_2_ pressure method (PetO_2_) (23). VT_1_ which is sometimes referred to as “anaerobic threshold” can be used for exercise intensity recommendations (24) but may also have a prognostic value in Fontan patients (25, 26). The oxygen uptake efficiency slope (OUES) was determined by plotting $\dot{V}O_{2}$ (ml/min) against the logarithm of $\dot{V}E$ (ml/min) and calculating the slope of this linear relation through single regression analysis (21).

We defined heart rate reserve (HRR) as the difference between target HR of each patient and the respective peak HR achieved by this patient. Even though HRR is usually defined as the difference between resting heart rate and target heart rate, we chose to use this definition as we wanted to investigate how much of their target heart rate could be achieved by each patient.

### Cardiopulmonary exercise test

Before the cardiopulmonary exercise test all subjects underwent a physical exam to rule out any acute disease. Blood gas and lactate (ABL 800 FLEX, Radiometer GmbH, Germany) were measured at the beginning and end of the test.

All subjects were fitted with a HR monitor (Polar H7 Bluetooth Smart 4.0 HR sensor, Kempele, Finland) and a face-mask. Gas exchange was measured using stationary exercise equipment (Metalyzer, Cortex, Leipzig, Germany).

Cardiopulmonary exercise testing was performed as an incremental step test on a treadmill (COSMED T 170, COSMED, Italy). For the incremental running test of the Fontan patients, we used an individualized treadmill testing protocol. The starting speed was set according to the fitness of each patient either at 3 or 4 km/h and then increased by 1 or 2 km/h. Each step lasted 2 min. The steepness of the treadmill was set at 1%. For patients with substantial amounts of edema we used a modified Bruce protocol so that the patients would not have to run (27). All patients were verbally encouraged to exercise until exhaustion. All tests were undertaken by the same researchers. The purpose of using individualized test protocols was a similar exercise time within a time frame of 8–10 min. In our opinion, this allows for better comparability of the test results as we have stated before (21).

### Statistical analysis

Statistical analysis was performed using Microsoft Excel 2,000^®^ for data collection and SPSS 12.0^®^ (SPSS Inc., Chicago, IL). All measured values are reported as means and standard deviations. The Kolgomorov-Smirnov test was used to check for normal distribution. Homogeneity of variance was investigated using Levine's F-test. For normally distributed variables differences between the physically active Fontan patients and their matched healthy control group as well as the differences between physically active and non-active Fontan patients were assessed with unpaired *t*-tests, otherwise the Wilcoxon or the Whitney-Mann-U-tests were used. Statistical significance was set at *p* < 0.05.

The Pearson correlation coefficient was used to investigate univariate correlation between independent variables and $\dot{V}O_{2}peak$.

For comparison of ordinal and nominal variables we used the Fisher's exact-Test.

## Results

### Subjects

We were able to compare 24 Fontan patients with a history of physical activity (15 male and 9 female) with 20 Fontan patients who had not been physically active in the past (14 male and 6 female). The distribution of sex, systemic ventricle, medication with beta-blocker, amount of physical activity as a preschooler, schoolchild, adolescent and recent in hours per week, school-/work transport habits, and subjective exercise capacity are depicted in Table 1. Six patients were unable to define their fitness level. These are the only missing data points. The anthropometric description including the z-score values for the children and adolescents of the two groups are represented in Table 2. There were no significant differences between the two groups with respect to age, height, weight or body mass index (BMI).

**Table 1:** Distribution of sex, systemic ventricle, medication with beta-blocker, school-/work transport habits and subjective exercise capacity as absolute values and in % in brackets.

		**Active fontans**	**Inactive fontans**	***P*-value**
Sex	Male	15 (62.5%)	14 (70)	0.75
	Female	9 (37.5%)	6 (30)	
Systemic ventricle	Left	15 (62.5 %)	10 (50%)	0.33
	Right	9 (37.5%)	10 (50%)	
Medication with beta-blocker	Yes	3 (12.5%)	6 (30%)	0.26
	No	21 (87.5%)	14 (70%)	
Physical activity as a preschool child (hours/week)		2.5 (± 3.0)	0.1 (± 0.2)	<0.001
Physical activity as a schoolchild (hours/week)		4.5 (± 3.6)	0.3 (0.6)	<0.001
Physical activity as an adolescent (hours/week)		5.1 (±3.3)	0.2 (± 0.4)	<0.001
Recent physical activity (hours/week)		3.3 (± 2.5)	1.0 (± 1.6)	<0.001
School-/transport habits	Car/bus	14 (58.3%)	12 (60%)	0.20
	Other	7 (30%)	1 (5%)	
Subjective exercise tolerance	Comparable to healthy control	12 (50%)	3 (15%)	0.09
	Poorer than healthy control	11 (46%)	12 (60%)	

The *p*-values for the exact Fisher test are also included.

**Table 2:** Mean anthropometric measurements for the two groups with standard deviation in brackets and the *p*-value for the *t*-test between the two groups.

	**Active Fontans**	**Inactive Fontans**	***P*-value**
Age (years)	16.8 (± 7.0)	19.7 (± 9.6)	0.25
Weight (kg)	54.3 (± 21.8)	50.7 (± 20.6)	0.58
z-Score	−0.35 (± 1.17)	−1.62 (2.12)	0.10
Height (cm)	162.0 (± 3.8)	156.9 (± 22.7)	0.47
z-Score	−0.10 (± 1.51)	−1.74 (± 2.34)	0.07
BMI (kg/m^2^)	19.7 (± 3.6)	19.6 (± 3.6)	0.90
z-Score (f/m)	−0.33 (± 1.16)	−0.53 (± 1.10)	0.71

BMI, body mass index.

### Biomarkers

We found no significant differences regarding hemoglobin, creatinine, glutamic oxaloacetic transaminase (GOT) or gamma glutamyl transferase (γ-GT) (Table 3). However, the value for N-Terminal-Pro-B-Type Natriuretic Peptide (NT-proBNP) was significantly lower in the group of active Fontans compared to the inactive ones.

**Table 3:** Laboratory results represented as means with standard deviation in brackets and the *p*-value for the *t*-test between the two groups.

	**Active Fontans**	**Inactive Fontans**	***P*-value**
NT-proBNP (mg/dl)	141.3 (± 108.5)	378 (± 293.8)	0.01
Hemoglobin (g/l)	16.0 (± 1.7)	15.3 (± 2.7)	0.30
Creatinine (mg/dl)	0.67 (± 0.22)	0.75 (± 0.24)	0.32
GOT (U/l)	31.8 (± 5.0)	28.4 (± 6.5)	0.06
γ-GT (U/l)	53.2 (± 37.6)	108.1 (± 143.9)	0.10

NT-proBNP, N-Terminal-Pro-B-Type Natriuretic Peptide; GOT, glutamic oxaloacetic transaminase; γ-GT, gamma glutamyl transferase.

### MRI evaluation

**Figure 1:**
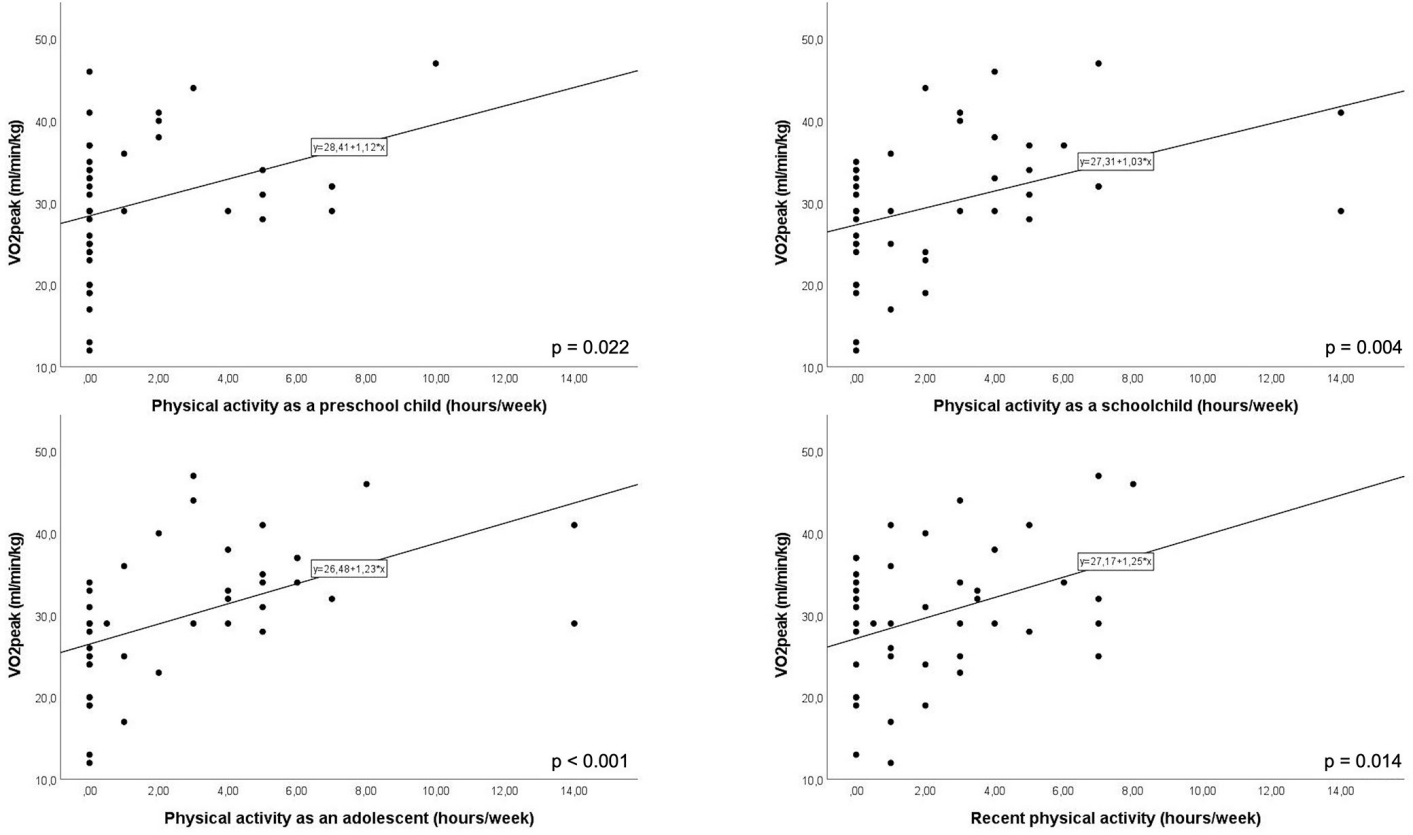
Correlation between hours of physical activity per week as a preschool child, as a schoolchild, as an adolescent and recently. Each of these correlations reached significance (*p*-values for each correlation are depicted in the lower right-hand corner).

The parameters measured in the MRI are presented in Table 4. There were no significant differences between the two groups with respect to aortic/neoaortic regurgitation, AV-valve regurgitation or EF (Figure 1). Furthermore, the number of patients with a low ejection fraction (EF <40%) was comparable between the two groups (four patients in the active group and two patients in the inactive group). Only one patient in the group of active Fontan patients and two in the group of inactive Fontan patients had aortopulmonary collaterals (AP collaterals). None had aortic or neoartic arch obstruction.

**Table 4:** MRI measurements as means with standard deviation in brackets and the *p*-value for the *t*-test between the two groups.

	**Active Fontans**	**Inactive Fontans**	***P*-value**
Amount of MRI's (%)	21 (87.5%)	17 (85%)	0.41
Time between CPET and MRI (months)	13.7 (± 19.3)	21.5 (± 25.6)	0.31
Number of patients with AP collaterals	2	1	0.42
Aortic regurgitation fraction (%)	1.4 (± 4.5)	5.8 (± 11.2)	0.24
AV-valve regurgitation fraction (%)	1.65 (± 6.3)	5.8 (± 11.2)	0.20
EF (%)	49.0 (± 9.9)	53.4 (± 10.7)	0.21

CPET, cardiopulmonary exercise testing; MRI, magnetic resonance imaging; AP collaterals, arterio-pulmonary collaterals; AV-valve, atrioventricular valve; EF, ejection fraction.

### *V̇O*_2peak_ and ventilatory threshold

The results from the cardiopulmonary exercise tests are represented in Table 5. The parameters for maximal exertion were comparable between both groups with a similar peak RER and peak lactate. One patient in the group of active Fontan patients did not achieve peak exertion with a RER <1.0. We only included measurements of submaximal exercise (OUES, $\dot{V}O_{2}$, HR and $\dot{V}E$ at VT1) for this patient. Even though the time to achieve $\dot{V}O_{2}peak$ (exercise time) was slightly longer in the group of active Fontan patients, this difference did not reach statistical significance. Both groups were well within the defined timeframe of 8–10 min for achieving peak exertion (RER >1.1).

**Table 5:** Mean values (± standard deviation) and the *p*-value for the *t*-test from the cardiopulmonary exercise test of active vs. inactive Fontans.

	**Active Fontans**	**Inactive Fontans**	***P*-value**
Peak RER	1.10 (± 0.08)	1.10 (± 0.07)	0.90
Resting lactate (mmol/l)	2.1 (± 0.6)	2.3 (± 1.0)	0.60
Peak lactate (mmol/l)	8.3 (± 3.1)	7.3 (± 1.3)	0.57
Exercise time (min)	9.3 (± 2.1)	8.0 (± 2.3)	0.06
	34.0 (± 7.1)	25.0 (± 6.6)	0.000
% of expected $\dot{V}O_{2}peak$	75.9 (± 25.1)	52.6 (± 23.0)	0.003
	56.0 (±9.4)	61.9 (± 10.0)	0.05
Peak heart rate (bpm)	169.8 (± 24.2)	139.8 (± 29.2)	0.001
Heart rate reserve (bpm)	11.4 (± 25.7)	39.5 (± 27.8)	0.001
Peak O_2_pulse (ml)	11.4 (± 5.4)	9.2 (± 5.0)	0.16
Peak $\dot{V}E$	78.3 (± 36.9)	59.2 (± 30.3)	0.02
PaPet O_2_ end	54.8 (± 9.1)	56.3 (± 17.1)	0.8
PaPet CO_2_ end	−4.2 (± 3.3)	−5.9 (± 4.1)	0.36
OUES	2.26 (± 1.11)	1.37 (± 0.83)	0.008
	34.3 (± 5.1)	43.9 (± 10.2)	0.000

PeakRER, Respiratory Exchange Ratio at the point of maximal exertion; $\dot{V}O_{2}peak$, peak oxygen uptake; VE, expiratory volume; PaPet O2 End, difference between arterial and endtidal O2-pressure at the end of exercise; PaPet CO2 End, difference between arterial and endtidal CO2-pressure at the end of exercise; OUES, oxygen uptake effiency slope; VE/VCO2-slope, correlation between expiratory volume to the volume of CO2.

$\dot{V}O_{2}$ achieved at peak exercise ($\dot{V}O_{2}peak$) differed significantly between the two groups with active Fontan patients achieving better values than non-active ones. The Fontan patients who had been physically active reached 75.9% of their predicted $\dot{V}O_{2}peak$ whereas the physically inactive patients only achieved 52.6%. This difference was equally significant.

$\dot{V}O_{2}$ at VT1 with respect to $\dot{V}O_{2}peak$ achieved (in %) was higher in the group of inactive Fontans compared to active ones.

### Cardiac function

The HR at peak exercise was also significantly higher for formerly active Fontan patients. As there was no significant difference between the two groups with respect to betablocker therapy, this difference seems not to be a consequence of medication (Table 1). The heart rate reserve (HRR) is the difference between the target peak HR and the actually achieved peak HR for each patient. Corresponding to the finding that the peak HR was higher in active Fontans, the HRR observed in constantly active Fontan patients is significantly lower than in constantly non-active Fontan patients (Table 5).

Peak O_2_-pulse, a surrogate parameter of stroke volume, did not differ between the groups.

There was no significant difference between the two groups for the O_2_-pulse, a surrogate parameter of stroke volume (Table 5).

### Pulmonary function

Even though the breathing frequency was similar for both groups did not differ between the two Fontan groups, the peak minute ventilation $\dot{V}Epeak$ was significantly higher in the group of physically active Fontan patients than in the ones who hadn't participated in sports (Table 5).

There was no significant difference between endtidal and arterial O_2_ (PaPetO_2_) as well as endtidal and arterial CO_2_ (PaPetCO_2_) at peak exercise between the two groups (Table 5).

### Slopes

The investigated slopes and results are summarized in Table 5. The oxygen uptake efficiency slope, a parameter determined at submaximal exertion was significantly higher in the group of physically active Fontan patients reflecting the results of the $\dot{V}O_{2}$peak. The $\dot{V}E$/$\dot{V}CO_{2}$-slope as an indicator for heart failure was also significantly higher in non-active than in active Fontan patients.

### Impact of physical activity

The amount of sport performed during each period of life (as a preschool child, school child, adolescent, recently) differed significantly between the groups (Table 1). Furthermore, this applies for recent sport activities as well (Table 1).

There was a positive correlation between hours of physical activity as a preschool child, as a schoolchild, as an adolescent, and recent physical activity with $\dot{V}O_{2}$peak (Figure 1).

### Systemic left ventricle vs. systemic right ventricle

There were more patients with a systemic left ventricle in the active Fontan group compared to more systemic right ventricles in the non-active Fontan group (Table 1). Even though this difference did not reach significance, we evaluated the difference between systemic right and systemic left ventricle in a separate approach. There was no difference between these two groups with regards to age, gender or anthropometric variables. The patients relying on a systemic right ventricle achieved lower values with regards to physical activity, CPET values, ejection fraction (EF) determined by MRI and laboratory values, but the difference reached significance in only few parameters, namely $\dot{V}O_{2}$peak, maximum speed achieved, peak breath rate and NT-proBNP. There was also no significant difference with regards to the hours of sports performed.

## Discussion

Improved cardiopulmonary function is related to better outcomes among Fontan patients (3, 9) and cardiac rehabilitation can promote exercise tolerance in this patient group (28). Thus, a higher physical activity level in these patients should lead to a higher exercise tolerance. This study aimed at evaluating the association between habitual physical activity and cardiopulmonary exercise capacity.

Patient characteristics did not differ significantly between the two groups, particularly with regards to sex or systemic ventricle.

Only few patients in the entire cohort had a medication with beta-blockers. Even though there were slightly more patients with beta-blocker therapy in the inactive Fontan group, this difference did not reach statistical significance. Most patients used the bus or the car to get to school or work. This is in accordance with current observations in which active school transport habits of children from high-income countries are declining (29, 30). Interestingly, 50% of the patients in the active Fontan group perceived their physical exercise capacity as comparable to their healthy peers, whereas only 15% of the inactive ones felt comparably fit.

Both groups were comparable with respect to all their anthropometric variables.

There were no significant differences with regards to most of the laboratory parameters such as hemoglobin, creatinine, GOT, or γ-GT. This is in accordance with Kodama et al. (14) who also observed no difference in hemoglobin between sport participant and non-participant Fontans. NT-proBNP, a marker of increased myocardial stress and volume and/or pressure overload of the ventricle (31), was significantly lower in the group of active Fontan patients. There is an association between BNP and NYHA class in patients with congenital heart disease as well as between adverse outcomes and elevated NT-proBNP values in Fontan patients (31, 32). However, the NT-proBNP values reported in the literature differ widely (31) and were shown to be within a normal range in the majority of Fontan patients in a large outpatient study involving 510 patients (33). Recently, the role of biomarkers and adverse outcomes in young Fontans had been investigated by van den Bosch et al. (34). The authors concluded that especially NT-proBNP may have a role in the clinical follow-up and risk-stratification of patients after Fontan procedure as in their study NT-proBNP was strongly associated with all possible adverse events investigated [cardiac death, out of hospital cardiac arrest, heart transplantation (listing), cardiac reintervention (severe events), hospitalization, and cardioversion/ablation for arrhythmias] (35). The results from our study with significant lower NT-proBNP values in Fontan patients with good cardiopulmonary function corroborates these findings from van den Bosch et al. Whether or not NT-proBNP should be used for outpatient surveillance, especially in asymptomatic patients, is questionable (36) and needs to be examined further.

All test subjects reached maximal exertion on the treadmill with comparable peak RER and peak lactate values. They achieved this with a comparable duration of the exercise test within 8–10 min. This reflects the adequate choice of exercise protocol which was individualized according to the capabilities of each test subject.

The most important parameter when investigating cardiorespiratory fitness is $\dot{V}O_{2}peak$ (37). Former studies were able to demonstrate a strong independent correlation between $\dot{V}O_{2}peak$, decline of $\dot{V}O_{2}peak$ or $\dot{V}O_{2}$ as percentage of predicted $\dot{V}O_{2}peak$ and morbidity (3, 36, 38, 39), cardiac adverse events (38), transplantation or death (38, 39). Ohuchi et al. (3) were able to show that a gain in $\dot{V}O_{2}peak$ from childhood to adulthood, which they call a positive exercise capacity trajectory, leads to a better adult Fontan pathophysiology including better prognosis (3). Former studies proved that Fontan patients who were sports club participants or took part in moderate-to-vigorous sporting activities showed significantly higher $\dot{V}O_{2}peak$. The authors suggested that exercise habits may promote exercise tolerance by improving respiratory function in Fontan adolescents (14, 34). This thesis corresponds with the newly proofed fact that inactivity leads to lower $\dot{V}O_{2}peak\;$-values even in healthy children (40). Another aspect of reduced cardiorespiratory function in Fontan patients is their reduced muscle mass (11, 41). Several studies have been able to show an association between $\dot{V}O_{2}peak$, peak oxygen pulse, skeletal muscle mass and skeletal muscle index, especially with respect to lower extremity muscle mass (11, 41). These findings in the context of already decreased muscle mass and exercise capacity in Fontan patients underlines the importance of maintaining muscle mass by increasing the amount of physical activity (41). Our investigation focused on the effect of physical activity during the lifespan of Fontan patients and their effects on cardiopulmonary fitness. In this respect our data fortify previous studies (14) with a significantly higher $\dot{V}O_{2}peak$ in the group of active Fontan patients compared to the non-active ones. The absolute $\dot{V}O_{2}peak$values in our active group were 75.9% of their expected $\dot{V}O_{2}peak$ which is similar to previously recorded results (10). As we did not include measurements of muscle mass, we can only speculate on the peripheral mechanisms at play for reduced oxygen uptake in the skeletal muscle.

We also investigated $\dot{V}O_{2}$ at the first ventilatory threshold (VT1) with respect to $\dot{V}O_{2}peak$ achieved, expressed in percent. Higher values of $\dot{V}O_{2}$ at VT1 (in percent of $\dot{V}O_{2}peak$) have been observed in cardiac patients suffering from heart failure in previous studies (24). In our study the fitter Fontan patients reached their VT1 at a lower percentage of their $\dot{V}O_{2}peak$ which is suggestive of a better cardiac function. Determining VT1 also plays an integral part when prescribing exercise recommendations (24).

Former studies presented a tight connection between peak HR and mortality (3, 36, 38, 39). Most of these studies describe a lower submaximal and maximal HR in Fontan patients (42). Von Scheidt (43) has proposed that the lower HR of Fontan patients is due to injury from repeated heart surgery and disconnection of the caval veins. Following this theory, one would suspect that the limitation of the maximal HR worsens with the number of operations. In our cohort the number of operations in both Fontan groups was comparable and undertaken by the same team of surgeons. Claessen et al. (7) postulated that this reduced peak HR is not a consequence of a pathology of the sinoatrial node but of hemodynamics. Performing cardiac MRI during supine bicycle exercise to near maximal exertion, they were able to show that this HR reduction is a consequence of reduced ventricular filling. The consequent reduction in stroke volume causes an early plateau in cardiac output which renders a further increase in HR physiologically implausible. Our data supports this hypothesis with the fact that there was a significant difference regarding peak HR between the two groups with active Fontan patients almost reaching their target heart rate and inactive Fontan patients achieving significantly lower peak heart rates. Hedlund et al. (42) postulated that the inability to further increase stroke volume and consequently cardiac output represented an important limiting factor for maximal cardiac output. Thus, being able to maintain a comparably high HR for physically active Fontan patients could represent the ability to generate a sufficient stroke volume even at higher HR, a feat impaired in less active peers.

Interestingly, we could not detect any difference in the ejection fraction determined with MRI between the two groups in our study. Even though the ventricle is the driving force for the circuit, it cannot compensate for the major restriction of the neoportal system created in the Fontan circulation, namely the pressure in the pulmonary veins (44). The ventricle no longer controls the cardiac output, and it has minor influence on the congestion in the veins. As a consequence of being a systemic single ventricle, it is likely that with time, systolic and diastolic function will deteriorate to some extent.

The patients in our cohort may have been too young to show this development. In accordance with the comparable EF, the O_2_pulse at peak exercise, a surrogate parameter of stroke volume at peak exercise, was also not significantly higher in the group of active Fontan patients compared to inactive ones. In Fontan patients the passive pulmonary circulation limits their ability to increase pulmonary blood flow and ventricular filling, leading to a lower O_2_ pulse, or stroke volume, during peak exercise (13, 16). This phenomenon seems to be more pronounced in the group of inactive Fontan patients, as the peak HR was significantly lower in the group of inactive Fontans. This could indicate limited capacity to keep ventricular filling adequate at higher HR.

Main pulmonary dysfunction in Fontan patients is shaped by restrictive ventilatory impairment (45) which, in turn, limits exercise capacity and even prognosis (17, 46). Patients with chronic heart failure improve their ventilatory efficiency by training of their inspiratory muscles (47). Ohuchi et al. (17) pointed out that there is a close association of pulmonary function and aerobic exercise capacity in Fontan patients and stressed the importance of exercise training for better pulmonary function. Our data confirm an impaired pulmonary function in the group of inactive Fontan patients with a significantly lower $\dot{V}Epeak$ at a comparable breathing frequency to the group of active Fontan patients.

The $\dot{V}E$ /$\dot{V}CO_{2}$-slope is also believed to have a prognostic value for heart failure in Fontan patients (3). This assumption is based on the close association between a steeper slope and an increased dead space due to the low compliance of the congested lung (3). Former studies have reported a correlation between $\dot{V}E$ /$\dot{V}CO_{2}$-slope and unscheduled hospitalization and morbidities (3, 36, 38). The significantly steeper $\dot{V}E$/$\dot{V}CO_{2}$-slope (higher values) in inactive Fontan patients stresses the importance of an active lifestyle for future positive long-term outcomes in Fontan patients.

What was surprising to us was the positive correlation between hours of physical activity and $\dot{V}O_{2}peak$ during all periods of the patient's life. Even early physical activity as a preschool child was positively correlated with $\dot{V}O_{2}peak$ in our study. This finding points out the importance of starting physical activity as early as possible allowing for a positive pediatric exercise capacity trajectory (17). This will lead to a better Fontan physiology in later life (17). However, it needs to be stressed that physical activity was only evaluated using a questionnaire leading to a possible recall bias with respect to the amount of physical activity performed.

As this was a retrospective study we cannot discern whether the physical activity was the reason for the better cardiopulmonary capacity or whether a better cardiopulmonary capacity of the Fontan patients led to being more active. However, since there was a positive correlation between hours spent being active during early childhood with $\dot{V}O_{2}peak$, we believe that it's the physical activity which led to a better cardiopulmonary capacity, as an impairment of cardiopulmonary capacity is at least less likely in this age group.

Other limitations of this study include the relatively small number of Fontan patients having been tested and the fact that this study was retrospective. Even though, the difference between systemic ventricle was not significant between the two groups, there were more systemic right ventricles in the inactive group than in the active one. The causes of this discrepancy cannot be explained by this retrospective study. Further studies are needed to evaluate specifically the benefit of physical activity for systemic right ventricles.

There is also the possibility that the performance of the inactive Fontan patients was not due to their physical inactivity but rather a consequence of a poor baseline hemodynamic condition with a low EF. As this is a retrospective study, it cannot be determined which came first, the poor cardiac function or the inactivity. However, the number of patients with a low EF was comparable in both groups.

The time between the most recent MRI and the cardiopulmonary exercise test was longer than 24 months in some patients. Therefore, the EF at the moment of cardiopulmonary exercise testing may have been worse. Still, 26 of the MRI's included in this study were <24 months apart from the CPET and only 8 were older than 36 months.

## Conclusion

$\dot{V}O_{2}peak$ a positive prognostic indicator was significantly higher in active Fontan patients, while the $\dot{V}E$ /$\dot{V}CO_{2}$-slope, a negative prognostic indicator was significantly lower in Fontan patients who had been physically active while growing up than in those who had been continuously inactive. The higher HR achieved by the physically active Fontan patients at maximal exertion imply the ability to maintain stroke volume at higher HR. The inability to further increase stroke volume and thus cardiac output has been suggested representing an important limiting factor for maximal cardiac output in Fontan patients.

Although this study is only retrospective in nature it points toward the fact that physical activity during childhood or adolescence could have an influence on prognostic values such as $\dot{V}O_{2}peak$, highlighting the importance of incorporating physical activity in the standard of care when treating Fontan patients.

## Data availability statement

The raw data supporting the conclusions of this article will be made available by the authors, without undue reservation.

## Ethics statement

The studies involving human participants were reviewed and approved by Ethikkommission der Friedrich-Alexander-Universität Erlangen-Nürnberg. Written informed consent to participate in this study was provided by the participants' legal guardian/next of kin.

## Author contributions

AW, RF, and IS conception and design of the study, data acquisition, data analysis and interpretation, drafting of manuscript, critical revision of manuscript, accountability for all aspects of work, and ensuring integrity and accuracy. KR conception and design of the study, data acquisition, data analysis and interpretation, critical revision of manuscript, accountability for all aspects of work, and ensuring integrity and accuracy. WW conception and design of the study, data acquisition, critical revision of manuscript, accountability for all aspects of work, and ensuring integrity and accuracy. JM, SD, and AP conception and design of the study, critical revision of manuscript, accountability for all aspects of work, and ensuring integrity and accuracy. All authors contributed to the article and approved the submitted version.

## Conflict of interest

The authors declare that the research was conducted in the absence of any commercial or financial relationships that could be construed as a potential conflict of interest.

## Publisher's note

All claims expressed in this article are solely those of the authors and do not necessarily represent those of their affiliated organizations, or those of the publisher, the editors and the reviewers. Any product that may be evaluated in this article, or claim that may be made by its manufacturer, is not guaranteed or endorsed by the publisher.
